# Supplementary material for: Early Measurement of Blood sST2 Is a Good Predictor of Death and Poor Outcomes in Patients Admitted for COVID-19 Infection
Source: J Clin Med. 2021 Aug 11;10(16):3534. doi: 10.3390/jcm10163534 (PMC8396994; doi:10.3390/jcm10163534)
Supplement: Supplementary file 1 [file jcm-10-03534-s001.zip › jcm-1318746-supplementary.pdf]

Supplementary Material

Early Measurement of Blood sST2 is a Good Predictor of Death and Poor Outcomes in Patients Admitted for COVID-19 Infection

Sánchez-Marteles M, Rubio-Gracia J, Peña-Fresneda N , Garcés-Horna V, Gracia-Tello B, Martínez-Lostao L, Crespo-Aznárez S, Pérez-Calvo JI ,Giménez-López I

SUPPLEMENTARY METHODS

Quantification of circulating sST2 in serum of hospitalized COVID-19 patients

Serum sST2 concentrations were determined in 150 COVID-19 patients admitted at hospital. Blood samples were withdrawn at admission, control and discharge. Blood was collected into clotting gel test tubes, centrifuged and serum was aliquoted and stored (Aragón's Health System Biobank) at -80ºC until analysis. All biological samples were collected under informed consent. Eventually, 144 admission and control, and 80 discharge samples were processed and analyzed. Serum aliquots were virus-inactivated by treatment with 1% Triton-X100. On the day of the analysis, serum was thawed and diluted 1:50 in Reagent Diluent buffer (1% Bovine Serum Albumin in Phosphate Buffered Saline). Serum concentrations of soluble ST2 were determined by sandwich enzyme-linked immuno-sorbent assay (ELISA), and following instructions provided by kit manufacturer (DY523B, R&D Systems Europe Ltd). Briefly, serum samples and standards were incubated with capture antibody adsorbed to 96 well plates, followed by incubation with detection antibody. Analyte-antibody complexes were detected using a Horseradish Peroxidase-based colorimetric assay and OD450 signal was recorded using a microplate reader. sST2 serum concentrations were calculated from standard curve after blank subtraction. A set of sera from 60 healthy donors obtained through Aragón's Health System Biobank (BSSA) was also analyzed in this manner. These sera had been originally collected from two independent sources, and were selected to match patient cohort age and gender distribution. Random samples from COVID patients and healthy donors were re-run in independent assays to test and correct for inter-assay variability.

SUPPLEMENTARY FIGURES

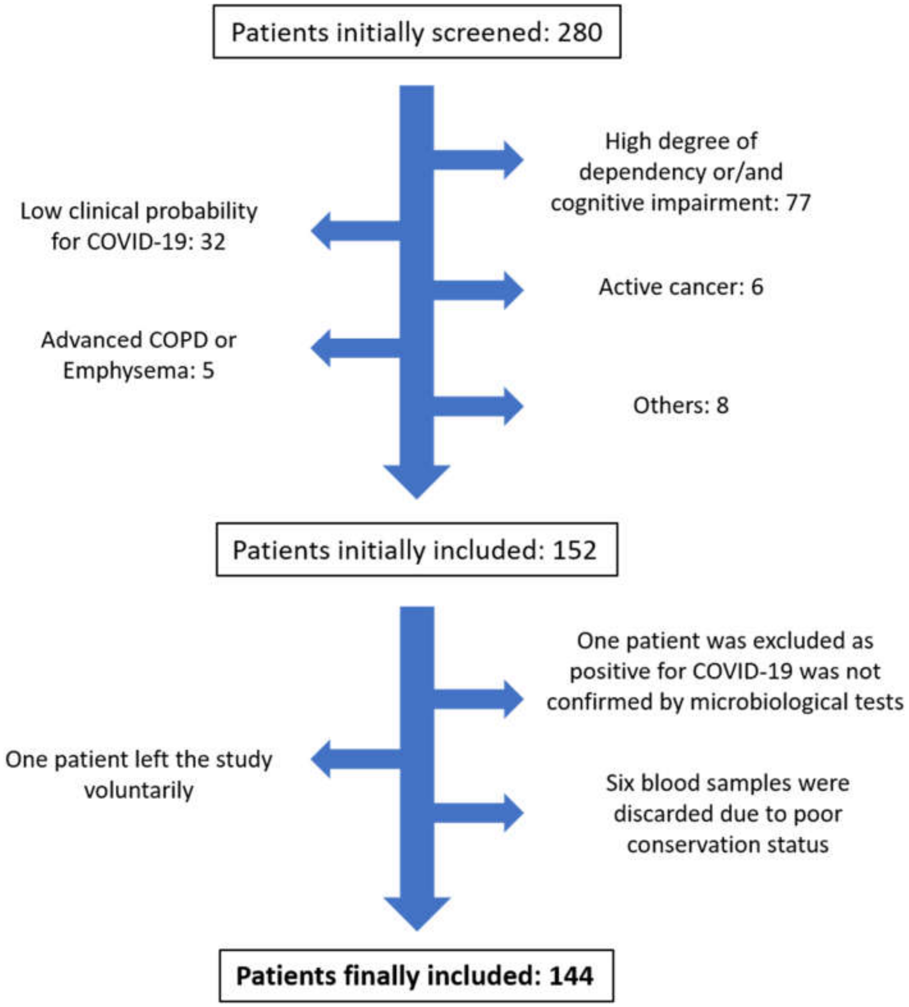

Supplementary Figure S1. Inclusion Flow chart.

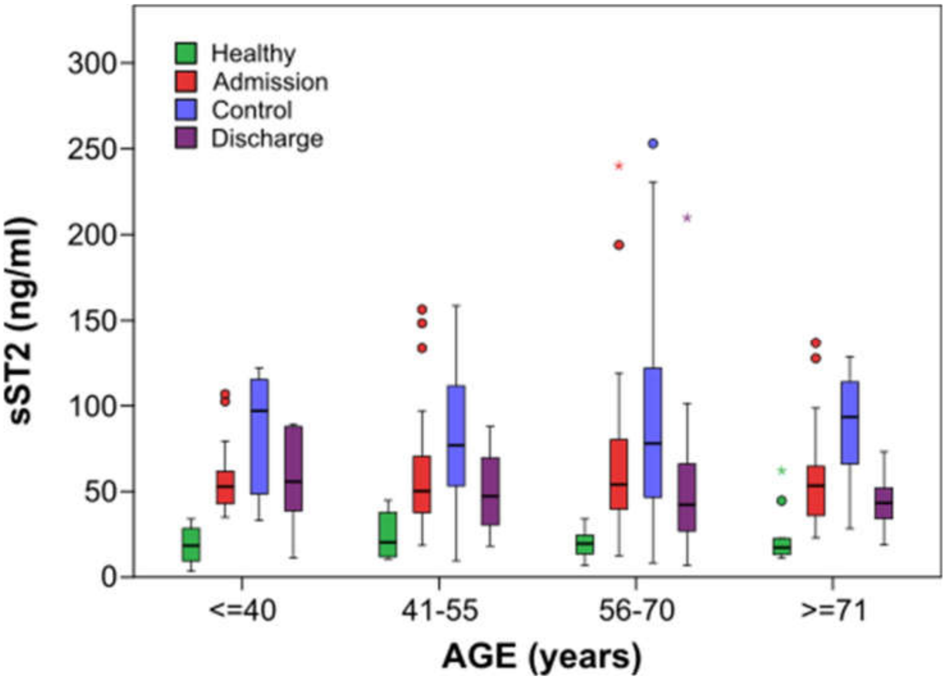

Supplementary Figure S2. sST2 concentrations across age groups. There were no differences between age groups in healthy donor, or COVID-19 patients (p>0.05).

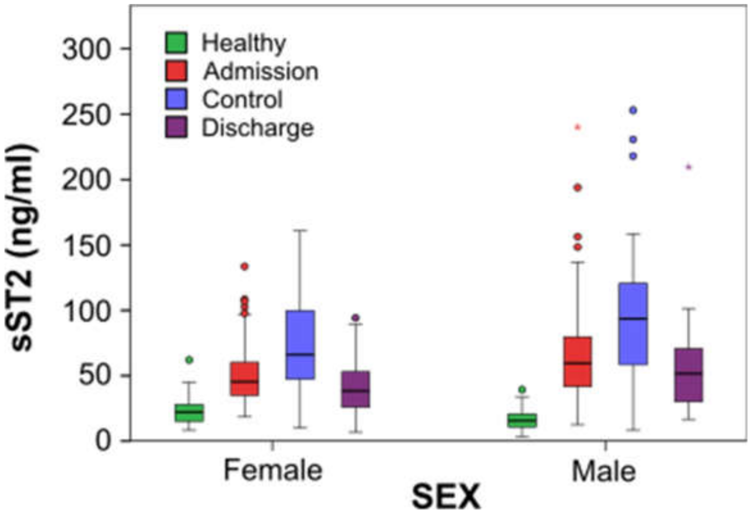

Supplementary Figure S3. Effect of gender on sST2 concentrations in COVID-19 disease. sST2 concentration was higher in female healthy donors than male (p=0.024). However, for COVID-19 patients, sST2 concentrations were higher in males vs females (p= 0.012).

SUPPLEMENTARY TABLES

Supplementary Table S1. Baseline characteristics according to primary outcome (in-hospital death and/or ICU admission for mechanical ventilation).

Variable	TOTAL	NO	YES	p Value
**Total size (N)**	144	129 (89.6)	15 (10.4)	
**Age (years)**	57.5 ± 12.8	56.8 ± 12.9	63.2 ± 10.4	0.069
**Gender-Male (n[%])**	87 (60.4)	79 (61.2)	8 (53.3)	0.553
**Duration of symptom (days)**	6.5 ± 3.3	6.5 ± 3.3	6.5 ± 2.6	0.974
**Time until COVID confirmation (Days)**	3 (7)	3 (7)	3 (6)	0.745
**Comorbidities (n[%]):**				
• Hypertension	54 (37.5)	46 (35.7)	8 (53.3)	0.181
• Heart failure	4 (2.8)	4 (3.1)	0 (0.0)	0.487
• Dyslipidemia	42 (29.2)	34 (26.4)	8 (53.3)	**0.030**
• Coronary artery disease	5 (3.5)	4 (3.1)	1 (6.7)	0.475
• Diabetes	25 (17.4)	20 (15.5)	5 (33.3)	0.084
• History of smoking	48 (33.6)	42 (32.8)	6 (40.0)	0.577
• COPD/Asthma	16 (11.1)	13 (10.1)	3 (20.0)	0.247
• Atrial/flutter fibrillation	5 (3.6)	4 (3.2)	1 (7.1)	0.448
• CKD	7 (4.9)	6 (4.7)	1 (6.7)	0.731
**Clinical variables**				
• BMI (Kgs/m2)	28.9 (6.4)	28.7 (6.9)	29.7 (4.9)	0.074
• SBP (mmHg)	126.9 ± 16.7	126.9 ± 17.1	127.2 ± 13.2	0.937
• DBP (mmHg)	77.2 ± 10.9	77.5 ± 11.2	74.9 ± 6.8	0.368
• HR (bpm)	80.9 ± 12.8	80.7 ± 12.8	82.1 ± 12.8	0.695
• Estimated PAFI (mmHg)	367 (92)	403 (88)	340 (103)	**0.005**
• Borg scale for dyspnea (points)	4 (6)	4 (6)	5 (4)	0.322
**Laboratory:**				
• Urea (mg/dL)	33 (19)	33 (20)	35 (20)	0.631
• Creatinine (mg/dL)	0.94 (0.29)	0.89 (0.29)	1.03 (0.31)	0.059
• Aspartate transaminase (U/L)	37 (27)	36 (27)	42 (29)	0.229
• Alanine transaminase (U/L)	31 (28)	31 (29)	29 (22)	0.955
• Creatin phophokinase (U/L)	94 (92)	92 (85)	142 (196)	0.227
• Lactate deshidrogenase (U/L)	306 (145)	297 (117)	382 (232)	0.069
• C-Reactive Protein (mg/L)	63 (81)	58 (74)	91 (128)	0.201
• Ferritin (ng/mL)	707 (908)	679 (933)	721 (478)	0.810
• Hemoglobin (g/dL)	14.2 ± 1.5	14.2 ± 1.53	14.0 ± 1.6	0.598
• Total leucocytes (x 1000)	5.6 (3.1)	5.5 (3.2)	6.9 (2.8)	0.397
• Total lymphocytes (x 1000)	0.9 (0.7)	0.9 (0.6)	0.7 (0.4)	0.136
• D-Dimer (ng/mL)	688 (633)	672 (648)	819 (680)	0.274
• Fibrinogen (mg/dL)	775 (208)	774 (217)	785 (155)	0.819
• Interleukine-6 (pg/mL)	40 (30)	39 (30)	48 (49)	0.389
• sST2 (ng/mL)	53.1 (30)	52 (28)	80 (49)	**0.001**
**X-Rays (n[%])**				0.696
• Normal	25 (17.9)	23 (18.4)	2 (13.3)	
• Unilateral consolidation	35 (25.0)	31 (24.8)	4 (26.7)	
• Bilateral consolidations	80 (57.1)	71 (56.8)	9 (60.0)	
**Therapies (n[%])**				
• Colchicine	10 (6.9)	9 (7.0)	1 (6.7)	0.964
• Plasma	1 (0.7)	1 (0.8)	0 (0.0)	0.732
• Remdesivir	46 (31.9)	41 (31.8)	5 (33.3)	0.903
• Systemic corticosteroids	113 (78.5)			

Variable	TOTAL	NO	YES	p Value
• Medium dose of corticosteroids (Dexametasone [mg])	6 (3)			
• Low molecular weight heparin	138 (95.8)	123 (95.3)	15 (100)	0.058

Variables are expressed as mean ± standard deviation or median (Interquartile range) BMI: Body Mass Index; CKD: Chronic Kidney Disease (estimated glomerular filtration rate < 60 mL/min/173m2 CKD-EPI-Creatinine method); COPD: Chronic Obstructive Pulmonary Disease; DBP: Diastolic Blood Pressure; HR: Heart Rate; SBP: Systolic Blood Pressure. sST2: Soluble ST2

Supplementary Table S2. Outcomes by sST2 concentrations at admission.

Variable	TOTAL	sST2 < 58.9	sST2 > 58.9	p Value
Primary outcome (n[%]):				
• ICU admission and/or death	15 (10.4)	3 (3.7)	12 (19.4)	**0.002**
Secondary outcomes:				
• Length of stay (days)	8 (6)	7 (5)	7 (5)	0.810
• Necessity of higher O2 therapy at 48/72 hours (n[%])	47 (34.1)	23 (29.1)	25 (40.7)	0.156
• Necessity to update COVID-19 treatment at 48/72 hours (n[%])	53 (37.9)	26 (32.9)	27 (44.3)	0.170
• Necessity of higher O2 therapy or update COVID-19 treatment at 48/72 hours (n[%])	66 (48.5)	33 (42.9)	33 (55.9)	0.131

ICU: Intensive Care Unit. ***Bold results*** *are statistically significant.*
